# Systemic neutralization of IL-17A significantly reduces breast cancer associated metastasis in arthritic mice by reducing CXCL12/SDF-1 expression in the metastatic niches

**DOI:** 10.1186/1471-2407-14-225

**Published:** 2014-03-27

**Authors:** Lopamudra Das Roy, Mahnaz Sahraei, Jorge L Schettini, Helen E Gruber, Dahlia M Besmer, Pinku Mukherjee

**Affiliations:** 1 Department of Biology, University of North Carolina at Charlotte, 9201 University City Blvd, Charlotte, NC 28223, USA; 2 Department of Pharmacology, Yale University, 10 Amistad St, New Haven, CT 06519, USA; 3 Caris Life Science, 4610 S 44th Place, Phoenix, AZ 85040, USA; 4 Department of Orthopedic Surgery, Carolinas Medical Center, Cannon Research Center, Room 304, PO Box 32861, Charlotte, NC 28232, USA

**Keywords:** CXCR4 CXCL12, SDF-1, Bone metastasis, Lung metastasis, Breast cancer, Autoimmune arthritis, IL-17A

## Abstract

**Background:**

IL-17A is a pro-inflammatory cytokine that is normally associated with autoimmune arthritis and other pro-inflammatory conditions. Recently, IL-17A has emerged as a critical factor in enhancing breast cancer (BC)-associated metastases. We generated immune competent arthritic mouse models that develop spontaneous BC-associated bone and lung metastasis. Using these models, we have previously shown that neutralization of IL-17A resulted in significant reduction in metastasis. However, the underlying mechanism/s remains unknown.

**Methods:**

We have utilized two previously published mouse models for this study: 1) the pro-arthritic mouse model (designated SKG) injected with metastatic BC cell line (4T1) in the mammary fat pad, and 2) the PyV MT mice that develop spontaneous mammary gland tumors injected with type II collagen to induce autoimmune arthritis. Mice were treated with anti-IL-17A neutralizing antibody and monitored for metastasis and assessed for pro-inflammatory cytokines and chemokines associated with BC-associated metastasis.

**Results:**

We first corroborate our previous finding that *in vivo* neutralization of IL-17A significantly reduced metastasis to the bones and lungs in both models. Next, we report that treatment with anti-IL17A antibody significantly reduced the expression of a key chemokine, CXCL12 (also known as stromal derived factor-1 (SDF - 1)) in the bones and lungs of treated mice. CXCL12 is a ligand for CXCR4 (expressed on BC cells) and their interaction is known to be critical for metastasis. Interestingly, levels of CXCR4 in the tumor remained unchanged with treatment. Consequently, protein lysates derived from the bones and lungs of treated mice were significantly less chemotactic for the BC cells than lysates from untreated mice; and addition of exogenous SDF-1 to the lysates from treated mice completely restored BC cell migration. In addition, cytokines such as IL-6 and M-CSF were significantly reduced in the lung and bone lysates following treatment. The data presented suggests that systemic neutralization of IL-17A can block the CXCR4/SDF-1 signaling pathway by reducing the expression of SDF-1 in the metastatic niches and significantly reducing metastasis in both mouse models.

**Conclusion:**

In our model, neutralization of IL-17A regulates SDF-1 expression in the metastatic niches either directly or indirectly via reducing levels of IL-6 and M-CSF.

## Background

By the year 2013, an estimated 750,000 women will die from breast cancer (BC) worldwide. Ninety percent of these deaths will be due to metastatic disease [1]. The most common site of metastasis is the bones and the lungs. Metastatic BC especially bone disseminated BC remains incurable [2]. Metastasis is regulated not only by intrinsic genetic changes in malignant cells, but also by the host microenvironment. Several studies have demonstrated that sites of chronic inflammation are often associated with establishment and growth of various types of malignancies [2,3]. A common inflammatory condition in humans is autoimmune arthritis (AA) with inflammation and deformity of the joints and increased cellular infiltration and inflammation in the lungs [4]. Several underlying molecular processes that characterize AA are also associated with cancer progression and metastasis. Epidemiologic studies indicate that BC patients with AA have poor prognosis and higher mortality rate compared to BC patients without AA [5]. To understand the molecular mechanisms and factors that facilitate BC-associated metastasis in arthritic conditions we have generated couple of models: one in which the mice are pro-arthritic and are later challenged to develop metastatic BC; and second in which the mice develop spontaneous BC first and then induced to develop arthritis [6-8]. Both mouse models are immune competent and develop significant levels of bone and lung metastasis. It is important to note here that without arthritis, very few mice develop lung and bone metastasis. Thus, these mice are ideally suited to study bone and lung metastasis that develops from the primary mammary gland tumors.

Using these models, we have previously reported that the inflammatory microenvironment caused by AA serves as a chemo attractant for recruitment, retention, and proliferation of BC cells in the bones and lungs [6-8]. We have identified interleukin-17A (IL-17A) as one of the critical pro-inflammatory cytokines within the metastatic niche and in the circulation, contributing to the enhanced metastasis. IL-17A mediates its pro-inflammatory effects by stimulating the release of multiple other cytokines such as IL-6, IL-8, and G-CSFs from epithelial, endothelial, and fibroblastic cells [9]. IL-17A is also associated with increased angiogenesis, proliferation, and metastasis in breast and other cancers [6,7,10]. Blocking IL-17A with a neutralizing antibody significantly reduced BC associated metastasis in both models [6,7].

In this study, we investigated the underlying mechanism/s of how systemic neutralization of IL17A inhibits/reduces metastasis. We report that IL-17A neutralization is highly effective in down- regulating the expression of CXCL12/SDF-1 in the metastatic niches. This in turn blocks the migration of the CXCR4-positive BC cells towards the metastatic niches. Data presented here shows that treatment with anti-IL-17A antibody significantly reduces metastasis to the bone and lungs in the two arthritic-BC mouse models by regulating the CXCR4/SDF-1 axis necessary for metastases. The study is of high significance since therapeutic strategies to block the CXCR4/SDF-1 interaction is being actively pursued [11-13].

## Methods

### Ethics statement

All experimental procedures were conducted according to Institutional Animal Care and Use Committee (IACUC) guidelines and the IACUC Committee of University of North Carolina at Charlotte (UNCC) has specifically approved this study (IACUC ID: 08–036.0 and 11–015.0). All mice were bred and maintained in specific pathogen-free conditions.

### Cell lines

The 4 T1 cells were purchased from The American Type Cell Culture Collection (Manassas, VA). Cells were maintained in complete RPMI [6]. The 4 T1 cells had stable expression of green fluorescent protein (4 T1-GFP). 4 T1 tumors is known to resemble human late stage metastatic BC [14-16]. The PyV MT cell lines were generated from PyV MT tumors and cultured as previously described in complete DMEM [7].

### Mice and treatment schema

SKG mice have been established from a closed breeding colony of Balb/C mice [17,18]. To test the efficacy of anti-IL-17A antibody treatment on BC associated metastasis, three month old SKG mice were injected with 1 × 10^5^ 4 T1-GFP cells (in 100 μl of PBS) in the mammary fat pad. When the tumors were ~5 mm in size (post ~6 days of 4 T1 injections), four intraperitoneal (ip) injections of 5 μg/ml of anti-IL17 antibody (Cat#560268; BD Pharmingen, San Diego, CA, USA;) or rat IgG isotype control antibody (Cat# 554682, BD Pharmingen) was administered once a week. 1XPBS was used as the solvent. Untreated 4 T1-tumor bearing SKG mice served as controls. The mice were euthanized 24 hours after the last injections at ~35 days post tumor challenge.

PyV MT oncogenic mice were originally a gift from Dr. W. J. Muller (McGill University, Toronto, Canada) [19]. The PyV MT mice were bred to be congenic on the C57BL/6 background and have been used in several of our prior publications [7,20-23]. At 12 weeks of age, PyV MT mice were injected intradermally with 50 uls of 2 mg/ml CII (Cat#804002-Lyo, MD Biosciences, St. Paul, MN, USA) in CFA (Difco laboratories, Michigan, USA) approximately 1.5cms distal from the base of the tail. Approximately 60% of mice develop arthritis within 15–30 days post collagen injection [24,25]. Four weeks post CII injection (at 16-weeks of age), four i.p injections of 5 μg/ml of anti-IL17 antibody or control IgG antibody (Cat#560268; BD Pharmingen, San Diego, CA) once every two weeks was administered. The mice were euthanized 24 hours after the last injections at ~23 weeks of age. Thus, mice received a total of 4 injections of the antibody. Untreated PyV MT mice served as controls.

#### Measurement of SDF-1 and IL-17A levels

SDF-1/CXCL12 levels were measured in the lung and bone lysates by a specific mouse Elisa kit (Cat# CKM061, Cell Sciences, Canton, MA). IL-17A levels were determined using a specific mouse Elisa kit (Cat#88-7371; eBioscience, San Diego, CA, USA). The limit of detection for the mouse CXCL12/SDF-1 Elisa kit from Cell Sciences, Cat#CKM061 is 9.38 pg/ml to 600 pg/ml. The limit of detection for the mouse IL17A ELISA kit from eBiosciences, Cat#88-7371 is 4 pg/ml to 500 pg/ml. 300 ug of tumor, lung and bone whole tissue lysates were used and manufacturer recommended protocols were followed. Results are expressed as picograms/ml. **Lysate preparation:** Briefly, lung tissue was collected in complete lysis buffer (20 mmol/L HEPES, 150 mmol/L NaCl, 1%Triton X100, and 2 mmol/L EDTA) supplemented with serine protease inhibitor (Complete inhibitor cocktail; Roche, Indianapolis, IN) and phosphatase inhibitor cocktail (Sigma-Aldrich,Missouri, USA). The tissues were homogenized using the IKA T25 digital ultra Turrax homogenizer (IKA, Wilmington, USA). After centrifugation, the supernatant was collected and used as the source of the protein lysate. Bones were collected in the same buffer and stored at -80°C. When ready to isolate protein, bones were placed on dry ice, cleared of all surrounding soft tissue and pulverized in liquid nitrogen using a mortar and pestle. The powdered bone was further homogenized in complete lysis buffer, centrifuged at 13000 rpm for 10 minutes and supernatant collected. A standard BCA assay was used to determine protein concentration (Cat#23225, Pierce BCA protein assay kit, Thermo Scientific, Rockford, USA).

### Antibodies for western blotting

CXCR4 antibody was used at a concentration of 1ug/ml (Cat#ab2074, Abcam, Cambridge, MA 02139) and secondary donkey anti-rabbit IgG HRP at 1:5000 dilution (Cat#sc-2313, Santa Cruz Biotechnology, Santa Cruz, CA). β-actin (Cat#sc-47778) antibody was used at 1:500 dilution and secondary anti-mouse IgG HRP (Cat#sc-2314) at 1:2000 dilution (Santa Cruz Biotechnology, Santa Cruz, CA). β-actin expression was used to confirm equal protein loading on the SDS-PAGE gels.

### Histology

Lung and bone sections were processed as previously described [6,7]. Paraffin embedded blocks were prepared and 4-micron thick sections were cut for hematoxylin eosin (H&E), SDF-1, and pancytokeratin staining. The SDF-1 (Cat#sc-74271) and pancytokeratin antibodies were purchased from Santa Cruz Biotechnology and used at 1:50 dilution following the same protocol as described in our previous publications [6,7] followed by DAKO anti-mouse secondary antibody (Cat# P0447, 1:100 dilution; Dako North America, Carpinteria, CA, USA).

### Image acquisition and analysis

All bright field images were taken using the Olympus DP71 light microscopy with Olympus BX60 camera (U-ND25-2, Olympus; Melville, NY) at magnifications shown in the figure legend. The images were analyzed by Caresbio Laboratory, Shelton, CT, USA. Image analysis algorithms were applied to the images generated from microscopic slides of tissues stained with DAB and hematoxylin. An algorithm was applied to the Red-Green-Blue (RGB)-filtered grayscale values from images. Images were analyzed using the image analysis software, MATLAB (R2011b, MathWorks). This provides the option for separation of DAB only- and double-stained areas from hematoxylin only- stained areas by means of subject specific thresholding. A good separation of DAB- and double-stained pixels from hematoxylin-stained pixels was achieved. Automatic background subtraction was performed applying median filters only. Significant differences in relative areas stained and mean specific intensity for the stains between control and treatment groups in mouse tissue were calculated.

### Invasion assays

4 T1 or PyV MT cells in serum free media (50,000 cells) were plated over transwell inserts (BD Biosciences, San Jose, CA), pre-coated with reduced growth factor matrigel (BD Biosciences, San Jose, CA, USA) and were permitted to invade towards lung and bone lysates (500 ug protein) contained in the bottom chamber for 24 hours. Percent invasion was calculated as absorbance of samples/absorbance of controls × 100 [6,7,26,27]. We added neutralizing anti-CXCR4 antibody (Cat#247506, R&D Systems, Minneapolis, MN) to tumor cells at 10ug/ml or recombinant mouse SDF-1 to the lower chamber at 0.6 ng/ml (Cat#460-SD, R&D Systems, Minneapolis, MN) and analyzed the invasion of BC cells.

### X-Ray imaging

The Pix array 100 x-ray machine (Bi Optic Inc, Santa Clara, CA, USA) was used for bone imaging as previously described [6-8,28].

### Measurement of cytokines

The RayBio® Custom Mouse Cytokines Antibody Array kit was purchased from Ray Biotech (Norcross, GA, USA) and used according to the manufacturer’s instructions. This is an immunoblot-based kit. The array of cytokines included in the study template was: IL-17A, IL-6, M-CSF, TNF-α, IGF-II, IL-4, IL-1B, Pro-MMP9, VEGF and osteoprotegerin. To measure the cytokines in the lung and bone microenvironment, 300ug of protein lysate was used. The densitometric analyses of immunoblots were performed using NIH Image J software (obtained from the NIH Web site: http://rsb.info.nih.gov/nih-image). Results are presented as mean values of arbitrary densitometric units corrected for background intensity and normalized using pre-determined controls (provided by the manufacturer).

### Statistical analysis

Data were analyzed using the GraphPad software. Results are expressed as mean ± s.e.m and are representative of greater than or equal to 3 replicate experiments. We used a one-way ANOVA with Tukey to compare all groups to each other. Comparison of groups was done by using 2-way ANOVA followed by the Bonferroni posttest for multiple comparisons. Student’s t-test was used for comparing the level of significance between two experimental groups.

**Figure 1:**
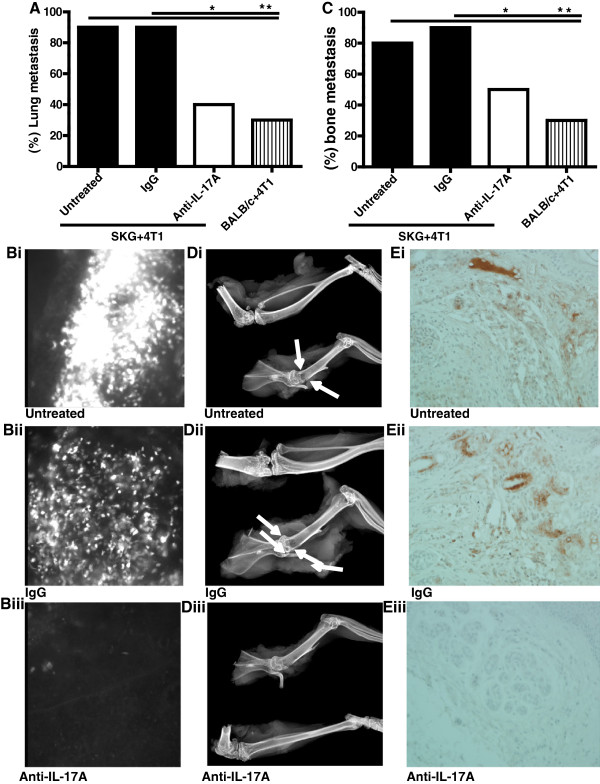
**Treatment with anti-IL17A significantly reduced lung and bone metastasis in SKG mice orthotopically injected with GFP positive 4 T1 BC cells. A**: Percentage of lung metastasis in 4 T1 tumor-bearing SKG mice ± treatment and in 4 T1 tumor-bearing non-arthritic Balb/C mice. Statistical test based on a normal approximation: The difference between untreated and IgG treated versus anti-IL-17 treated is significant (*p = 0.006) and difference between untreated SKG + 4 T1 versus Balb/C + 4 T1 is also significant (**p = 0.001). **Bi-iii**: Representative images of GFP positive 4 T1 cells in lungs with no treatment (i), control IgG antibody treatment (ii) or anti-IL17A antibody treatment (iii). **C**: Percentage of bone metastasis in 4 T1 tumor-bearing SKG mice ± treatment and in 4 T1 tumor-bearing non-arthritic Balb/C mice. Assuming normal approximation, the difference between IgG control and anti- IL-17 treated group is significant (*p = 0.03) and difference between untreated SKG + 4 T1 and Balb/C + 4 T1 is also significant (**p = 0.001). Significance was not reached between untreated SKG + 4 T1 and IL-17A treated group although lower numbers of mice developed bone metastasis with anti-IL-17A antibody treatment. **Di-iii**: Representative X-ray images of metastatic bone lesions in 4 T1 tumor bearing SKG mice with no treatment (i) 4 T1 tumor bearing SKG mice treated with control IgG antibody (ii) or anti-IL-17A antibody treatment (iii) Arrows point to a metastatic site in the proximal humerus; note the prominent lucency in the proximal region, reflecting extensive bone loss at this site . (A-D: N = 10 mice). **Ei-iii**: Representative images of pancytokeratin staining of bone tissue to confirm metastasis in 4 T1 tumor bearing SKG mice with no treatment (i) 4 T1 tumor bearing SKG mice treated with control IgG antibody (ii) or anti-IL-17A antibody treatment (iii).

**Figure 2:**
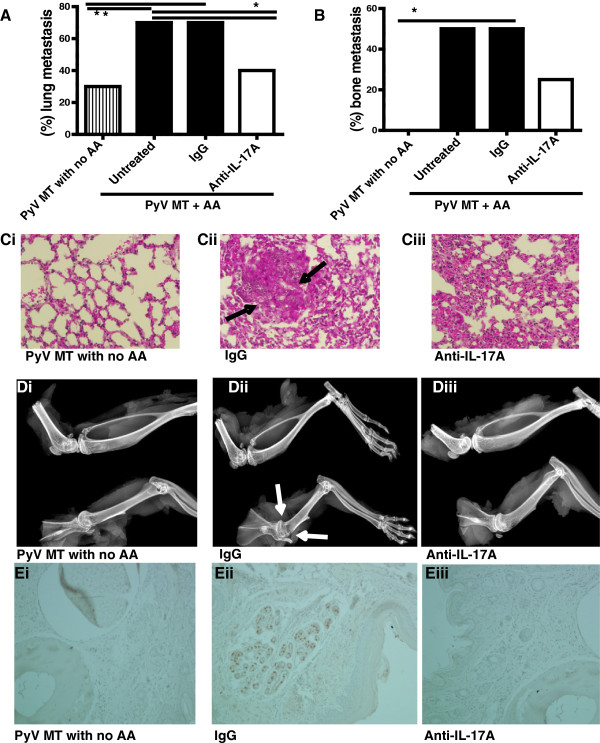
**Diminution of lung and bone metastasis in arthritic PyV MT mice treated with anti-IL-17A antibody. A** and **B**: Percentage of lung **(A)** and bone **(B)** metastasis in PyV MT mice Statistics: The difference in lung metastasis between untreated and IgG treated versus anti-IL-17 treated is significant (*p = 0.006) and difference between untreated PyV MT + AA versus PyV MT with no AA is also significant (**p = 0.001). Although fewer animals developed bone metastasis with anti-IL-17 antibody treatment, significance was not reached. However there was a significant difference in bone metastasis between PyVMT mice with no AA versus PyVMT mice with AA (*p = 0.002) **C**: Representative images of H& E (200x magnification) of lungs to confirm metastasis in PyV MT mice with no AA (i), PyV MT mice with AA treated with control IgG antibody (ii) or with anti-IL-17A antibody (iii). **D**: Representative X-ray images of metastatic bone lesions in PyV MT mice with no AA (i); arthritic PyV MT model treated with control antibody (ii) or anti-IL-17A antibody (iii) (A-D: N = 6–8 mice) Arrows point to a metastatic site in the proximal humerus; note the prominent lucency in the proximal region, reflecting extensive bone loss at this site. **Ei-iii**: Representative images of pancytokeratin staining of bone tissue to confirm metastasis in PyV MT mice with no AA (i), PyV MT mice with AA treated with control IgG antibody (ii) or with anti-IL-17A antibody (iii).

For Figure 1A and C and Figure 2A and B, Minitab was used to conduct the statistical test based on a normal approximation for percentage differences.

## Result

### Significant reduction in metastasis in the 4 T1- tumor bearing SKG mice and the arthritic PyV MT mice treated with anti-IL-17A antibody

We selected two models to test the efficacy of the anti-IL-17A antibody treatment on BC-associated bone and lung metastasis: 1) the SKG mice challenged with 4 T1 tumors and 2) the PyV MT mice induced with CII. We selected only the arthritic models because very few non-arthritic tumor-bearing mice (0–3 mice out of 10) develop bone or lung metastasis as compared to their arthritic counterparts (5–9 mice out of 10) (Figures 1A and B & 2A and B).

In the 4 T1-tumor bearing SKG mice, data demonstrates a significant reduction in the percent of mice that develop bone and lung metastasis when treated with anti-IL17A antibody compared to control mice (80-90% of control mice develop metastasis versus only 40-50% of treated mice develop metastasis) (Figure 1A and C). Also shown are representative images of a) GFP positive lesions in the lungs (Figure 1B (i-iii)), b) radiographic metastatic lesions in the bone (Figure 1D (i-iii)), and c) pancytokeratin brown staining representing epithelial cell lesions in sections of bone confirming metastasis (Figure 1E (i-iii)). In addition, densitometric analysis was performed for pancytokeratin IHC images and the data is tabulated in Table 1. Arrows in Figure 1D (i and ii) point to a metastatic site in the proximal humerus; note the prominent lucency in the proximal region, reflecting extensive bone loss at this site.

**Table 1:** **Densitometry analysis of the pancytokeratin expression on the bones of the representative images from Figure **1** Ei-iii**

	**IntDen**	**StdDev**	**%Area**
SKG + BC + Untreated	2326.73	20.57	7.34
SKG + BC + IgG	3830.90	20.4	9.65
SKG + BC + anti-IL-17A	964.780*	18.67	1.19

(i) 4 T1 tumor bearing SKG mice with no treatment; (ii) 4 T1 tumor bearing SKG mice treated with control IgG antibody or (iii) with anti-IL-17A antibody (*P < 0.01: SKG + BC + anti-IL-17A against all other groups)

The effect of the anti-IL-17A antibody treatment on primary tumors showed a small but significant reduction in tumor size as previously published [6] and shown as Additional file 1: Figure S1A.

In the arthritic PyV MT mice, similar results were observed. Treatment with anti-IL-17A showed a significant decrease in the percent of mice that developed metastasis (Figure 2A and B). Data shows that ~70% (7 out of 10) of control mice developed lung metastasis and 50% (5 out of 10) developed bone metastasis (Figure 2A and B). When treated with anti-IL-17A antibody, only 40% (4 out 10) and 30% (3 out of 10) developed lung and bone metastasis respectively (Figure 2A and B). Representative H&E staining confirming metastatic lesion in the lung is shown in Figure 2Ci-iii while representative radiographic images of the bone lesions and pancytokeratin staining confirming metastasis in sections of the bone is shown in Figure 2Di-iii and Figure 2Ei-iii (for lung) and 4 (for bone). In addition, densitometric analysis was performed for pancytokeratin IHC images and the data is tabulated in Table 2.

**Table 2:** **Densitometry analysis of the pancytokeratin expression on the bones of the representative images from Figure **2** Ei-iiix**

	**IntDen**	**StdDev**	**%Area**
PyV MT with no AA	928.63*	20.46	0.73
PyV MT + CII + IgG	2188.64	19.78	14.09
PyV MT + CII + anti-IL-17A	889.44*	20.36	0.68

(i) PyV MT mice with no AA; (ii) PyV MT mice with AA treated with control IgG antibody or (iii) with anti-IL-17A antibody. (*P < 0.01: PyV MT and PyV MT + CII + anti-IL-17A against PyV MT + CII + IgG).

Treatment with anti-IL-17A antibody had no effect on the primary tumor burden in the arthritic PyV MT mice (Additional file 1: Figure S1B). Thus, the study focused on the effect of anti-IL-17A antibody treatment on metastasis.

### Significant decrease in the expression of pro-inflammatory cytokines in treated mice

**Figure 3:**
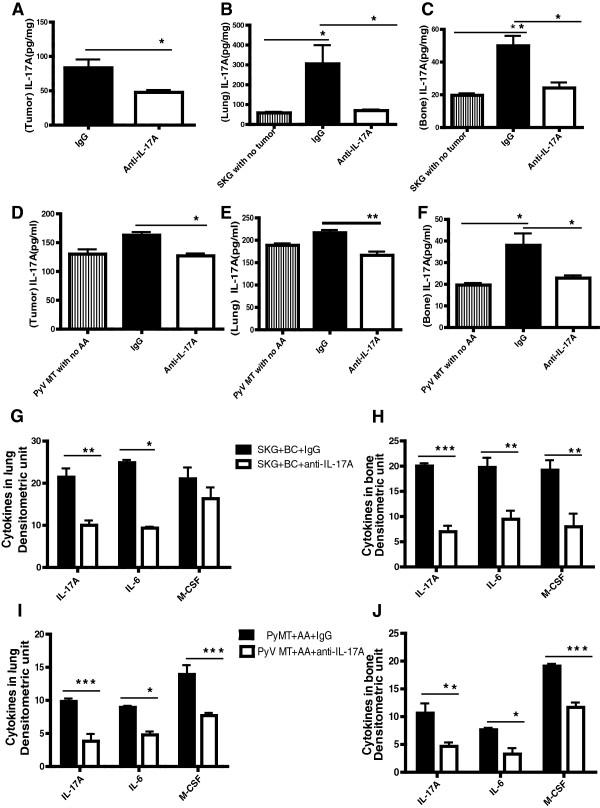
**Anti-IL17A treatment down regulates pro-inflammatory cytokines associated with IL-17A expression. A-C**: Levels of IL-17A (pgs/ml) by ELISA in the tumor, lung and bone protein lysates of SKG mice (*P < 0.05;**P < 0.01). **D-F**: Levels of IL-17A by ELISA in the tumor, lung and bone of PyV MT mice (**P < 0.01; *P < 0.05). The limit of detection for mouse IL-17A is 4 pg/ml to 500 pg/ml. **G-J**: Ray Biotech cytokine array densitometry values: **G** and **H**: 4 T1 tumor bearing SKG mice. **I** and **J**: Arthritic PyV MT mice (*P <0.05, **P < 0.01, ***P < 0.001). N = 4 mice.

To determine if treatment reduced IL-17A levels within the tumor and metastatic niches, we evaluated the levels of IL-17A using an ELISA. Compared to the control group, the level of IL-17A in the tumor of SKG mice treated with anti-IL-17A antibody was significantly lower (Figure 3A). Level of IL-17A in the lungs and bones was also significantly reduced as compared to control mice (Figure 3B and C respectively) with levels as low as seen in SKG mice with no tumors (Figure 3B and C). Similarly, we observed a significant decrease in IL-17A levels in the tumors, lungs and bones of the PyV MT mice treated with anti-IL-17A antibody compared to the control groups (Figure 3D, E and F respectively). Non-arthritic PyV MT mice served as another control.

Since IL-17A mediates its downstream effects by stimulating the release of multiple other cytokines [9], we examined which factors in the bone and lung may be influenced by anti-IL-17A antibody treatment. In both the SKG and PyV MT mice, multiple cytokine array analyses showed a significant reduction in the levels of IL-6 and M-CSF in addition to reduction in IL-17A levels in treated versus control mice (Figure 3G and H for SKG mice and Figure 3I and J for PyV MT mice). The array of cytokines included IL-17A, IL-6, M-CSF, TNF-α, IGF-II, IL-4, IL-1B, Pro-MMP9, VEGF and osteoporotegerin. None of the other cytokines showed significant differences.

### Reduced SDF-1 (CXCL12) expression in lungs and bones of treated mice

**Figure 4:**
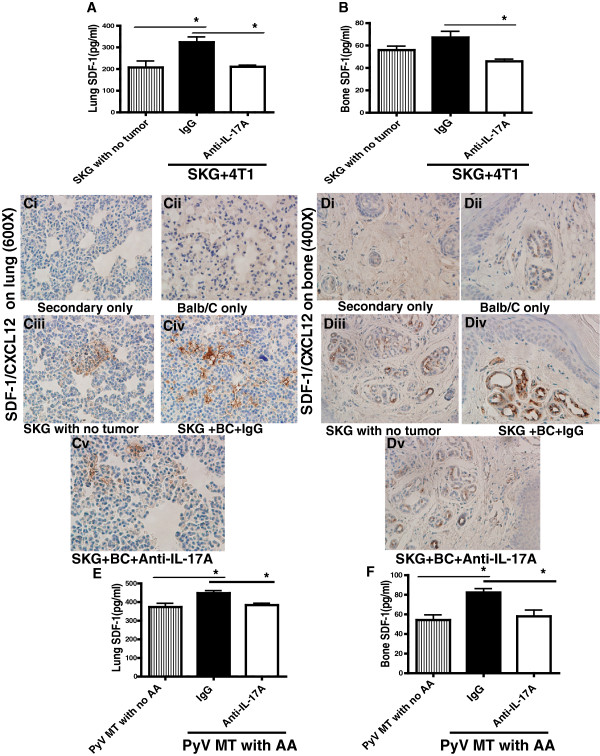
**Significant down-regulation of SDF-1 (CXCL12) expression in the metastatic niches. A** and **B**: Levels of SDF-1 by ELISA in the lung **(A)** and bone **(B)** lysate of SKG with no tumor, 4 T1 tumor bearing SKG mice treated with control or anti-IL-17A antibody (*P < 0.05) (N = 3 mice). The limit of detection for mouse SDF-1 is 9.38 pg/ml to 600 pg/ml. **C** and **D**: Representative images of SDF-1 expression by IHC in lung **(C)** and bone **(D)** (N = 6 mice), (i) Secondary antibody only; (ii) Balb/C with no tumor or AA (iii) SKG with no tumor; (iv) 4 T1 tumor bearing SKG mice treated with control IgG antibody or (v) with anti-IL-17A antibody. Brown staining represents SDF-1 localization (Bone at 400X magnification and Lung at 600X magnification). **E** and **F**: Levels of SDF-1 by ELISA in the lung **(E)** and bone **(F)** lysate of PyV MT mice with no AA and PyV MT + AA with control or anti-IL-17A antibody treatment (*P < 0.05) (N = 3 mice).

BC metastasis is known to be facilitated by the interaction of the chemokine SDF - 1/CXCL12 with its ligand CXCR4 [12]. Thus, we first investigated if anti-IL-17A antibody treatment affects the expression of SDF-1 in the metastatic niches. We observed a significant reduction in SDF-1 levels by specific ELISA in the lung and bone lysates of the treated versus control mice (Figure 4A and B respectively). Non-tumor bearing SKG mice were included in the study as control. Data clearly suggests that when SKG mice were induced to develop tumors, the level of SDF-1 significantly increased in the lungs and that treatment with anti-IL-17A antibody brought the levels back down to the non-tumor bearing levels (Figure 4A). Similarly, in the bones, the level of SDF-1 was significantly decreased with anti-IL-17A treatment (Figure 4B). Control non-arthritic Balb/C mice have low levels of SDF-1 (data not shown: 26.8 pg/ml and 64.11 pg/ml in the bone and lung lysate respectively). The data was further confirmed by immunohistochemistry (IHC) staining of lung and bone tissue sections. Representative images of lung and bone sections are shown in Figures 4 Ci-iii and Di-iii respectively. In addition, densitometric analysis was performed for all IHC images and the data is tabulated in Table 3 (for lung) and Table 4 (for bone). It is apparent from the data that non-arthritic Balb/C mice have low expression of SDF-1 (Figure 4Cii and Dii) while SKG mice without any tumor have elevated levels of SDF-1 as noted by ELISA (Figure 4A&B) and by IHC (Figure 4Ciii and Diii and Table 3). This suggests that SDF-1 expression increases with induction of arthritis prior to tumor development.

**Table 3:** **Densitometry analysis of the SDF-1 expression on the lungs of the representative images from Figure**4** Ci-v**

	**IntDen**	**StdDev**	**%Area**
Secondary negative control	32.97**	30.27	0.3
Balb/C with no tumor	1001.25*	26.59	1.23
SKG with no tumor	1154.60*	25.53	1.98
SKG + BC + IgG	4249.27	21.13	25.49
SKG + BC + anti-IL-17A	1179.99*	24.97	4.49

(i) Secondary antibody only; (ii) Balb/C with no tumor and no AA; (iii) SKG with no tumor; (iv) 4 T1 tumor bearing SKG mice treated with control IgG antibody or with anti-IL-17A antibody (v) (*P < 0.05: Balb/C, SKG, SKG + BC + anti-IL-17A against SKG + BC + IgG; **P < 0.01: Secondary antibody against all other groups).

**Table 4:** **Densitometry analysis of the SDF-1 expression on the bones of the representative images from Figure**4** D i-v**

	**IntDen**	**StdDev**	**%Area**
Secondary negative control	1040.48*	24.75	0.69
Balb/C with no tumor	1105.238*	26.41	1.15
SKG with no tumor	1432.77*	22.72	1.29
SKG + BC + IgG	2124.35	26.42	10.25
SKG + BC + anti-IL-17A	1250.82*	22.09	2.72

(i) Secondary antibody only; (ii) Balb/C with no tumor and no AA; (iii) SKG with no tumor; (iv) 4 T1 tumor bearing SKG mice treated with control IgG antibody or with anti-IL-17A antibody (v). (*P < 0.05: all groups against SKG + BC + IgG).

Similar results were observed in the PyV MT mice where the level of SDF-1 was significantly increased when mice were induced with CII to develop arthritis (Figure 4E and F). However, treatment with anti-IL-17A antibody completely reversed the effect of CII and reduced the level of SDF-1 to that of non-arthritic mice in the lung and bone (Figures 4E and F respectively).

To reconfirm that anti-IL-17A treatment indeed reduces the level of SDF-1 in the arthritic mice prior to the development of tumor, we treated non-tumor bearing arthritic mice (C57BL/6 mice injected with CII) with the anti-IL-17A antibody. We found that anti-IL-17A antibody treatment indeed reduces the CII-induced SDF-1 levels in the bone and lung prior to tumor development (Additional file 2: Table S1). The data therefore suggests that treatment with anti-IL-17A antibody reduces the level of SDF-1 first which subsequently reduces the migration of the CXCR4+ BC cells to the bones and lungs.

**Figure 5:**
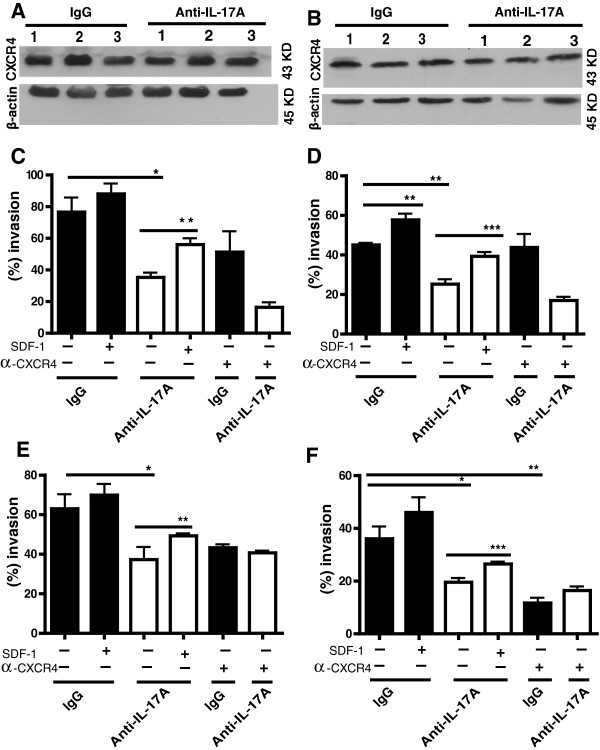
**Bone and lung lysates from mice treated with anti-IL-17A antibody are less chemotactic than their control antibody treated counterparts. A and B)** Western blotting showing the presence of CXCR4 on the tumors derived from **A)** 4 T1 tumor bearing SKG mice and **B)** PyV MT-arthritic mice treated with control or anti-IL-17A antibody (N = 3 tumors). **C** and **D**: In vitro trans-well invasion assay using 4 T1 cells in the upper chamber and lung **(C)** or bone **(D)** lysate from 4 T1 tumor bearing SKG mice treated with control or anti-IL-17A antibody in the lower chamber. **E** and **F**: In vitro trans-well invasion assay using PyV MT tumor cells in the upper chamber and lung **(E)** or bone **(F)** lysate from arthritic PyV MT mice treated with control or anti-IL-17A antibody in the lower chamber**.** Various treatment groups and significance are shown in the figure. *P < 0.05, **P < 0.01, ***P < 0.001 (N = 5 mice). We used a one way ANOVA with Tukey to compare all groups to each other.

Next, we assessed the expression of CXCR4 on the tumors dissected from the control and treated mice by western blotting. No change in the CXCR4 expression was observed in the 4 T1 or the PyV MT tumors with treatment (Figure 5A and B respectively). N = tumors from 3 individual mice are shown. We have also shown as Additional file 3: Figure S2, the entire western blot image of CXCR4 expression in tumors from Figure 5A and B. The appropriate size for CXCR4 is 43 KD shown in Figure 5.

### Treatment with anti-IL-17A antibody significantly reduces the chemotactic potential of lung and bone lysates for BC cells

It is well established that CXCR4^+^ cancers metastasize to the distant organs in a CXCL12/SDF-1-dependent manner [29-31]. Since we observed that the level of SDF-1 was significantly reduced with anti-IL-17A antibody treatment, we conducted an *in vitro* trans-well Boyden chamber assay with the bone or lung lysate in the bottom chamber and the 4 T1 or PyV MT tumor cells in the top chamber. There was a significant decrease in the migration of 4 T1 cells towards the lung (Figure 5C) and bone (Figure 5D) lysates derived from treated mice (Figure 5C and D bar# 3) as compared to the lysates derived from control mice (Figure 5C and D bar# 1). Similarly, migration of PyV MT tumor cells towards the lung (Figure 5E) and bone (Figure 5F) lysates from treated mice was significantly lower compared to migration towards control lysate (Figure 5E and F bar# 3 compared to bar #1).

Further, we demonstrate that addition of recombinant SDF-1 to the lung and bone lysates in the lower chamber reversed the effect of anti-IL-17A treatment and significantly increased the migration of the 4 T1 and PyV MT tumor cells towards the lower chamber (compare bar# 3 to bar# 4 in Figures 5C-F). Finally, we tested if blocking CXCR4 would have a similar effect. Data demonstrates that adding anti-CXCR4 neutralizing antibody to the 4 T1 and PyV MT tumor cells in the upper chamber had some effect on % migration, but in most instances the difference did not reach statistical significance (Figures 5C-E bar# 1 versus bar# 5, and Figures 5C-F bar# 3 versus bar# 6). However, in one instance, with PyV MT tumor cells treated with anti-CXCR4 antibody, there was a significant drop in % invasion towards bone lysate. (Figure 5F bar# 1 versus bar# 5).

Taken together our data suggests that in arthritic condition, IL-17A blockade reduces BC-associated metastasis by specifically reducing SDF-1 levels in the metastatic niches and thereby affecting their chemotactic potential.

## Discussion

Previously we established that the PyV MT mice that develop spontaneous mammary gland tumors develop severe bone and lung metastasis when induced with CII. If not induced with CII, these mice do not develop bone metastasis while 50% of CII induced PyV MT mice develop bone metastasis [6-8] and Figure 2B). Similarly, only 20-30% of PyV MT mice without CII develop lung metastasis but when induced with CII, ~80% of the mice present with lung metastasis [6-8] and Figure 2A. The primary tumors are also larger in the arthritic PyV MT mice [7]. Correspondingly, in the pro-arthritic SKG mice (which is in the Balb/C background), establishment of the 4 T1 tumors in the mammary fat pad gives rise to bone metastasis in 80-90% of the mice [6,8] and Figure 1B. In contrast, 30% of the Balb/C mice (which are not pro-arthritic) bearing the 4 T1 tumors develop bone metastasis [6,8] and Figure 1B. With regards to lung metastasis, 30% of 4 T1 tumor-bearing Balb/C mice develop lung metastasis while the same 4 T1 tumors generate lung metastasis in 90% of pro-arthritic SKG mice [6,8] and Figure 1A. The primary 4 T1 tumors are also larger in the SKG mice [6,8].

Using these unique arthritic models of BC metastasis, we previously established that neutralizing IL-17A can significantly reduce both bone and lung metastasis [6,7] and Figures 1 and 2. However, the underlying mechanism of action of IL-17A remained unknown.

Data clearly demonstrate that treatment with the anti-IL-17A antibody reduced the expression of SDF-1/CXCL12 in the bones and lungs (Figure 4). SDF-1 plays a critical role in the mobilization and recruitment of CXCR4+ BC cells to the neo-angiogenic niches supporting tumor growth and metastasis [32,33]. It is known that malignant primary BC cells express high levels of chemokine receptor CXCR4. When these cells pass through the organs that express large amounts of the chemokine SDF-1/CXCL12, the cells leave the circulation and enter the organs [11,34]. The CXCL12/CXCR4 axis is known to be involved in several aspects of tumor progression including angiogenesis, metastasis, and survival [30]. Our data is the first to show that in arthritic condition, blockade of IL-17A can disrupt this critical interaction of CXCR4 with SDF-1 by significantly reducing the SDF-1 levels in the bones and lungs and thus inhibiting the migration of the CXCR4+ BC cells towards the metastatic niches. Furthermore, inhibition of migration of BC cells was completely reversed by exogenously adding SDF-1 to the bone and lung lysate in an *in vitro* migration assay (Figure 5C - F). This suggests that the SDF-1 expression is critical for mobilizing the tumor cells to migrate.

It is also of interest that the downstream effect of IL-17A neutralization was a reduction in IL-6 and M-CSF (Figure 3G - J). This was to be expected as IL-17 is known to a) directly activate other immune cells to produce IL-6, IL-8, and PGE_2._[35] and b) indirectly activate the anti-inflammatory Th2 type cytokines including IL-10 and IL-13 that are known to reduce levels of IL-6 and M-CSF [35]. M-CSF is a cytokine involved in the development and proliferation of the monocyte/macrophage lineage cells and is reported to participate in the induction of osteoclasts, which is important in the destruction of bone and cartilage [36]. Thus, reduction of M-CSF with the anti-IL-17A antibody treatment supports the hypothesis that bone destruction due to arthritis creates a supportive milieu for BC cells to metastasize. Indeed in our previous publications, we have shown that BC-associated metastasis is significantly augmented in mice with arthritis and that IL-17A, IL-6, COX-2, VEGF, MMP-9, IGF-II, M-CSF and TNF-α are all major players [6,7]. These cytokines not only play an imperative role in arthritis but also cancer development and progression [37-48].

The emergence of IL-17A blockade as a future therapy in AA is already reported and initial observations from phase I trials show that signs and symptoms of AA are significantly suppressed following treatment with anti-IL-17A antibodies, without notable adverse effect [49]. Thus, we focused on IL-17A blockade and in understanding the underlying mechanism by which IL-17A blockade inhibits metastasis.

The experiments and the unique mouse models utilized in this study implicate the importance of targeting IL-17A for preventing metastasis associated with metastatic BC. Whether IL-17A blockade directly or indirectly (via reducing M-CSF and IL-6) regulates the SDF-1 levels needs to be deciphered and will be the focus of our next study. Nevertheless, data provides a rationale for designing potential therapies that may utilize IL-17A blockade in combination with conventional treatment regimen for patients with metastatic BC that also present with arthritis.

We recognize that other BC-bone metastasis models that do not have any arthritis should be tested in the future. However, to the best of our knowledge, there is currently no immune competent spontaneous BC-bone metastasis model available.

## Conclusion

We conclude that in our model, neutralization of IL-17A regulates SDF-1 expression in the metastatic niches either directly or indirectly via reducing levels of IL-6 and M-CSF.

## Competing interests

The authors declare that they have no competing interests.

## Authors’ contributions

LDR designed and carried out all the experiments, and wrote the manuscript. MS, JLS and DMB helped with the endpoints. MS helped with the ELISAs. HEG interpreted the x-ray imaging. PM is the principal investigator of the laboratory in which the research was performed and contributed to the interpretation of the data and writing of the manuscript. All of the authors have read and have approved the final manuscript.

## Authors’ information

Pinku Mukherjee, PhD, Irwin Belk Distinguished Professor of Cancer Research, Department of Biology, University of North Carolina, Charlotte, NC. Dr Mukherjee has worked on Breast Cancer for the past 22 years.

Lopamudra Das Roy, PhD, Research Director, CanDiag Inc and Adjunct Assistant professor, Department of Biology, University of North Carolina, Charlotte, NC. Dr Das Roy has received funding for her work in Breast Cancer Research from the US Department of Defense. Dr Das Roy has immense experience on research with Breast Cancer and Arthritis and received press releases determining the association between arthritis and Breast Cancer associated metastasis.

Mahnaz Sahraei, PhD, Post doctoral fellow, Department of Pharmacology, Yale University . Dr Sahraei graduated from University of North Carolina, Charlotte, NC under the mentorship of Dr Pinku Mukherjee.

Jorge L. Schettini, PhD, is a trained immunologist and worked with Dr Mukherjee for three years. Currently, he is working as a Research Scientist at Caris Life Sciences, Phoenix, AZ.

Dahlia M Besmer, PhD, graduated from Department of Biology, University of North Carolina, Charlotte, NC. DMB has received funding for her work on Breast Cancer from the US Department of Defense.

Helen E. Gruber, PhD, Director, Biology Division, Department of Orthopedic Surgery, Carolinas Medical Center, Charlotte, NC. Dr Gruber has over 28 years of experience in the area of bone pathology and osteoarthritis and bone metastasis.

## Pre-publication history

The pre-publication history for this paper can be accessed here:

http://www.biomedcentral.com/1471-2407/14/225/prepub

## Supplementary Material

Additional file 1: Figure S1 Kinetics of primary mammary gland tumor growth in arthritic mice with BC *±* treatment: A) SKG mice with 4 T1 tumors treated with anti-IL17A versus untreated or IgG control groups (*P < 0.05; **P < 0.01, ***P < 0.001); B) PyV MT mice with AA and treated with anti-IL17A versus untreated or IgG control groups. Click here for file

Additional file 2: Table S1 Level of SDF-1 in bone and lung lysate of non-tumor bearing normal and arthritic mice treated with anti-IL-17A antibody. Click here for file

Additional file 3: Figure S2 The entire western blot image of CXCR4 expression in tumors from Figure 5A and B. The appropriate size for CXCR4 is 43 KD shown in Figure 5. A) 4 T1 tumor bearing SKG mice treated with control IgG or anti-IL-17A antibody (N = 3 tumors); B) PyV MT-arthritic mice treated with control IgG or anti-IL-17A antibody (N = 3 tumors). Click here for file
