# Supplementary figures and images for: Systemic neutralization of IL-17A significantly reduces breast cancer associated metastasis in arthritic mice by reducing CXCL12/SDF-1 expression in the metastatic niches
Source: BMC Cancer. 2014 Mar 27;14:225. doi: 10.1186/1471-2407-14-225 (PMC3986611; doi:10.1186/1471-2407-14-225)

Supplemental Figure 1

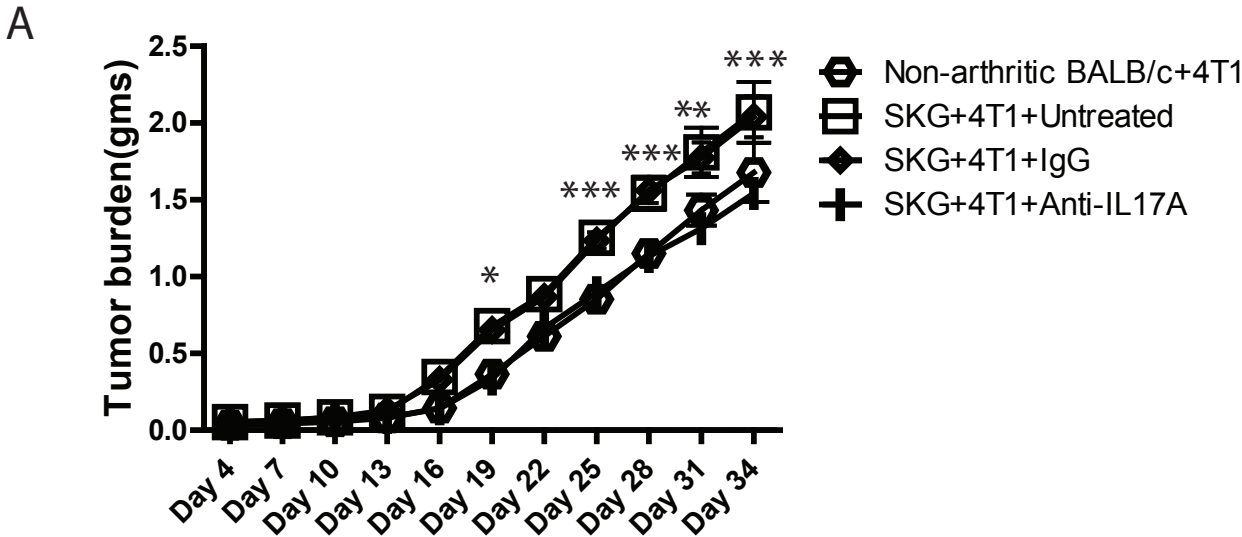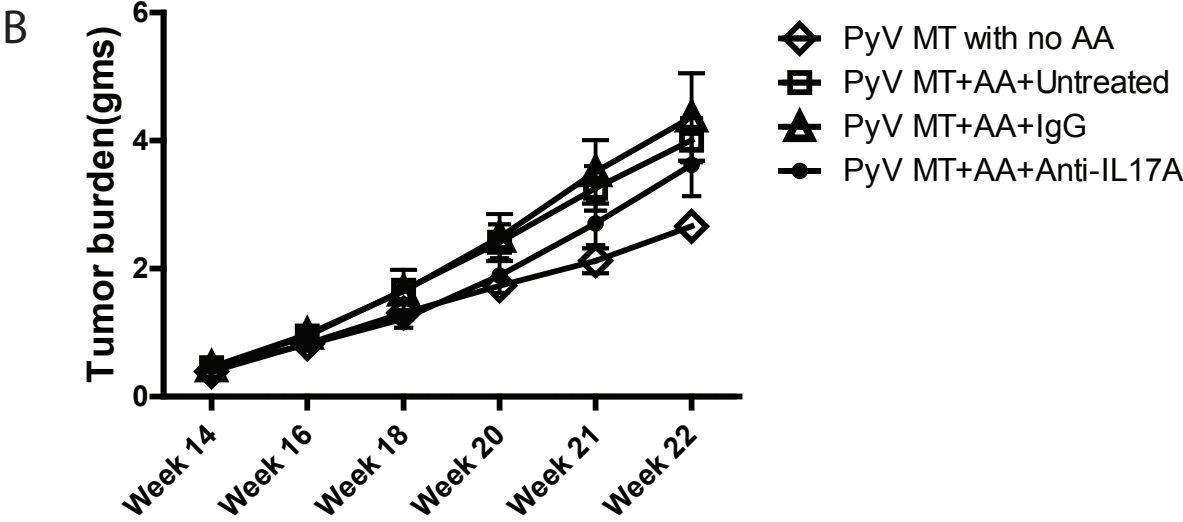

Supplement: Additional file 1: Figure S1 — Kinetics of primary mammary gland tumor growth in arthritic mice with BC ± treatment: A) SKG mice with 4 T1 tumors treated with anti-IL17A versus untreated or IgG control groups (*P < 0.05; **P < 0.01, ***P < 0.001); B) PyV MT mice with AA and treated with anti-IL17A versus untreated or IgG control groups. [file 1471-2407-14-225-S1.pdf]
