# Supplementary material for: Systemic neutralization of IL-17A significantly reduces breast cancer associated metastasis in arthritic mice by reducing CXCL12/SDF-1 expression in the metastatic niches
Source: BMC Cancer. 2014 Mar 27;14:225. doi: 10.1186/1471-2407-14-225 (PMC3986611; doi:10.1186/1471-2407-14-225)

Supplemental Figure 2

A CXCR4 from the tumors derived from 4T1 tumor bearing SKG mice

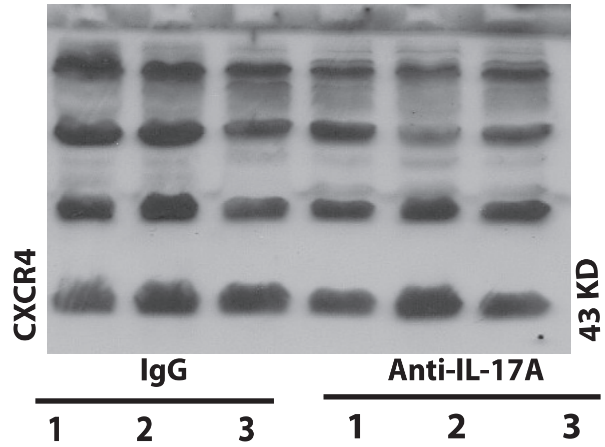

B CXCR4 from the tumors derived from PyV MT-arthritic mice treated with control or anti-IL17A antibody

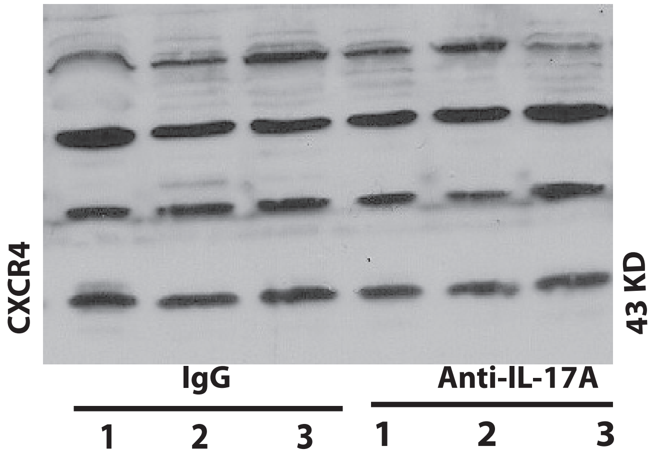

Supplement: Additional file 3: Figure S2 — The entire western blot image of CXCR4 expression in tumors from Figure 5A and B. The appropriate size for CXCR4 is 43 KD shown in Figure 5. A) 4 T1 tumor bearing SKG mice treated with control IgG or anti-IL-17A antibody (N = 3 tumors); B) PyV MT-arthritic mice treated with control IgG or anti-IL-17A antibody (N = 3 tumors). [file 1471-2407-14-225-S3.pdf]
